# Subgrouping by gene expression profiles to improve relapse risk prediction in paediatric B‐precursor acute lymphoblastic leukaemia

**DOI:** 10.1002/cam4.3842

**Published:** 2021-05-13

**Authors:** Qingsheng Huang, Jiayong Zhong, Huan Gao, Kuanrong Li, Huiying Liang

**Affiliations:** ^1^ School of Mathematics and Statistics Hanshan Normal University Chaozhou China; ^2^ Institute of Paediatrics Guangzhou Women and Children’s Medical Centre Guangzhou Medical University Guangzhou China; ^3^ State Key Laboratory of Ophthalmology Zhongshan Ophthalmic Center Sun Yat‐sen University Guangzhou China; ^4^ Clinical Data Center Guangdong Provincial People’s Hospital/Guangdong Academy of Medical Sciences Guangzhou China

**Keywords:** B‐precursor acute lymphoblastic leukaemia, gene expression profiles, minimal residual disease, non‐negative matrix factorization, relapse

## Abstract

Relapsed acute lymphoblastic leukaemia (ALL) remains a prevalent paediatric cancer and one of the most common causes of mortality from malignancy in children. Tailoring the intensity of therapy according to early stratification is a promising strategy but remains a major challenge due to heterogeneity and subtyping difficulty. In this study, we subgroup B‐precursor ALL patients by gene expression profiles, using non‐negative matrix factorization and minimum description length which unsupervisedly determines the number of subgroups. Within each of the four subgroups, logistic and Cox regression with elastic net regularization are used to build models predicting minimal residual disease (MRD) and relapse‐free survival (RFS) respectively. Measured by area under the receiver operating characteristic curve (AUC), subgrouping improves prediction of MRD in one subgroup which mostly overlaps with subtype TCF3‐PBX1 (AUC = 0·986 in the training set and 1·0 in the test set), compared to a global model published previously. The models predicting RFS displayed acceptable concordance in training set and discriminate high‐relapse‐risk patients in three subgroups of the test set (Wilcoxon test *p* = 0·048, 0·036, and 0·016). Genes playing roles in the models are specific to different subgroups. The improvement of subgrouped MRD prediction and the differences of genes in prediction models of subgroups suggest that the heterogeneity of B‐precursor ALL can be handled by subgrouping according to gene expression profiles to improve the prediction accuracy.

## Graphical abstract

Subgrouping of B‐precursor ALL patients by non‐negative matrix factorization of gene expression profiles improves prediction of minimal residual disease and relapse‐free survival. Such an improvement and differences in genes among subgroups suggest that the heterogeneity of B‐precursor ALL can be handled by subgrouping according to gene expression profiles.

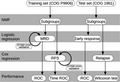

## 1 INTRODUCTION

Acute lymphoblastic leukaemia (ALL) is the most common paediatric leukaemia.^1^ Although progressive developments in chemotherapy and treatment intensity stratification based on risk evaluation considerably improve the survival rate, achieving 90% today,^2^ relapsed ALL remains a prevalent paediatric cancer and one of the most common cause of mortality from malignancy in children.^3^ Tailoring the intensity of therapy according to early identification of patients of high relapse risk is a promising strategy and also the major challenge.

Prognostic factors of paediatric ALL valuable to clinical decision include clinical features at diagnosis, subtypes defined by cell lineage (e.g. T‐ALL and B‐ALL) and genetics (e.g. TCF3‐PBX1, MLL, etc.), and early response to induction therapy.^2^ Genetic subtypes, deduced from immunophenotype, cytogenetic features and gene expression profiles indicate genetic alterations such as aneuploidy, indels of DNA segments, mutation and rearrangements of genes on the chromosomes.^2^ The alterations result in dysregulations of gene expression and abnormal proteins, which perturb key cellular processes and are associated with prognosis and drug resistance.^4^ Precise subtyping is critical for successful treatment.^5^ There are, however, exceptions that patients with a low‐risk genetic subtype (like ETV6‐RUNX1) are not cured, whereas patients with a high‐risk subtype (e.g. certain MLL rearrangements) are cured,^4^ suggesting patients of the same subtype can be further stratified. Immunophenotypic subtyping requires interpretation and integration of the complex patterns produced by flow cytometry, and no single marker is robust and sufficient to determine the subtypes^55^. Gene expression profiles correlate with genetic changes, and have been used to distinguish cell lineage and to identify some genetic subtypes.^4^ Bhojwani *et al*. developed logistic regression models to predict outcome by gene expression signatures with no genetic subtyping because genetic subtyping was impossible for patients lacking known subtypes in the cohort and could be unnecessary for prediction of outcomes.^6^

ALL is genetically polyclonal at first diagnosis. After induction therapy, proliferative predominant clones are suppressed or eliminated, but subclones acquiring mutations may survive if the cells resistance to specific chemotherapeutic agents.^2^ The level of minimal residual disease (MRD) at the end of induction therapy, as a measure of disease burden and therapeutic response, has been proved to be the most powerful indicator of relapse risk in paediatric ALL.^7^, ^8^ Based on the level of MRD at the end of induction therapy, intensification of therapy for high‐relapse‐risk patients improves the outcomes.^9^ However, waiting for the measurement of MRD by flow cytometry at the end of induction therapy precludes early intervention in high‐relapse‐risk patients.^9^ An earlier prediction of MRD positivity implying failed induction therapy will provide a further chance to tailor the induction method or to adopt other treatment interventions. Kang *et al*. developed MRD classifiers based on a 21‐gene signature in pre‐treatment blood or bone marrow specimens, which effectively substituted for MRD measurement at the end of induction therapy in prediction of relapse‐free survival (RFS).^9^

Paediatric ALL consists of various subtypes. Prognostic signatures may exist within biologic subtypes of ALL only. Risk stratification via subtyping often encounters embarrassment when patients lack known subtypes.^6^ Although gene expression profiles provide sufficient features to define novel subtypes, the sample size of a subtype is usually too small to prevent prediction models from over‐fitting because a large number of features introduce noise.^6^ Predicting outcomes by a gene signature globally for all subtypes is promising to overwhelm the problem of small sample size, but has been shown to be difficult for such a heterogeneous disease.^6^, ^10^ Balancing the non‐linearity of subtyping and the homogeneity of a gene signature appeals a framework for modelling outcomes with the heterogeneous data of paediatric ALL.

**FIGURE 1:**
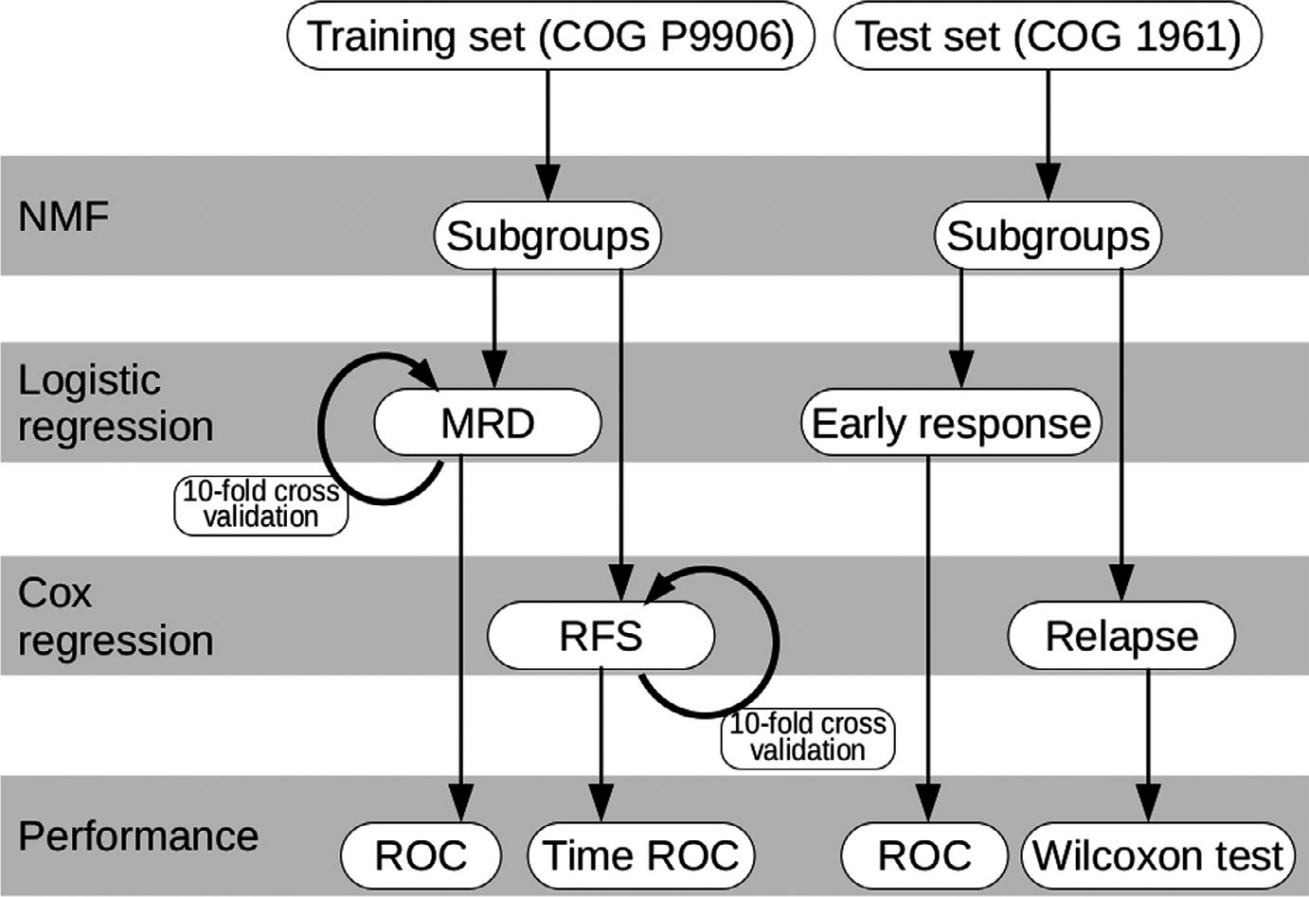
Flowchart of the prediction model combining NMF for subgrouping and logistic and Cox regressions for predicting MRD and RFS. Beside 10‐fold cross‐validations, performance of the model is validated by predicting the early response and relapse in the test set

In this study, we implement a combination of a non‐linear subgrouping step and a regression step to predict the outcome of paediatric B‐precursor ALL by gene expression profiles (Figure 1). Achieved by non‐negative matrix factorization (NMF), the subgrouping step mimics genetic subtyping, but does not aim at predicting strictly the gold‐standard subtypes. Instead, subgrouping splits the cohort into groups unsupervisedly to eliminate the heterogeneity of gene expression. Although some of the resulting subgroups overlap with the genetic subtypes, a subgroup that mixes with a few subtypes and even contains unknown subtypes is allowed. Within each subgroup, models predicting MRD and RFS are built by logistic regression and Cox regression with elastic net regularization respectively. The two‐step modelling improves the performance of prediction of MRD and RFS in subsets of the training and test cohorts. The gene sets identified by the logistic regression and Cox regression confirm the heterogeneity of B‐precursor ALL.

## 2 MATERIALS AND METHODS

### 2.1 Gene expression profiles and clinical records

We collected gene expression data and clinical records of two cohorts, namely the Children's Oncology Group Clinical Trial P9906 (COG P9906)^9^, ^11^ and the Children's Oncology Group 1961 (COG 1961).^6^ The trial of COG P9906 targeted high‐risk B‐precursor ALL patients (*n* = 207), including patients with central nervous system or testicular leukaemia, excluding patients with very high‐risk subtypes (BCR‐ABL1 or hypodiploidy) and excluding patients with low‐risk features (trisomies of chromosomes 4 or 10 and ETV6‐RUNX1, if no central nervous system or testicular leukaemia). The patients were treated uniformly with a modified augmented Berlin‐Frankfurt‐Münster Study Group (BFM) regimen. At the end of induction therapy (day 29), MRD of most patients was assessed by flow cytometry, where MRD positivity was defined by a threshold of 0·01%. RFS was recorded as the number of days from the trial enrolment to either the first event (relapse) or last follow‐up.

The COG 1961 study published gene expression data and clinical records of patients (*n* = 99) with high‐risk B‐precursor ALL, without subtypes predictive of outcome. The patients received a standard four‐drug induction therapy. The patients had bone marrow assessed on day 7, and were classified as slow early responders (M3, >25% blasts) or rapid early responder (M1, <5% blasts, and M2, 5% to 25% blasts). Long‐term outcome was characterized by the time of relapse, and patients were classified as in complete continuous remission (CCR) for at least 4 years and with marrow relapse within the first 3 years.

The study was performed in accordance with the Declaration of Helsinki, and do not publish information from human participants. We obtained the gene expression microarray data of COG P9906 and COG 1961 from NCBI Gene Expression Omnibus under the accession GSE11877 and GSE7440 respectively. We used the data of COG P9906 as training set, and the data of COG 1961 as test set (Table 1).

**TABLE 1:** Clinical features and outcomes in the studied cohorts

	**Training set**	**Test set**
Clinical trial	COG P9906^9^, ^11^	COG 1961^6^
GEO^a^ accession	GSE11877	GSE7440
**Sample size (*N*)**	207	99
**Age (year)**		
>10	132	61
<10	75	38
Median	13	11
Range	1–20	1–18
**Gender**		
Male	137	61
Female	70	38
**WBC (×10^3^ μL^−1^)**		
Median	62.3	65.8
Range	1.0–958.8	1.8–732.0
**Early response to therapy**	Day 29 MRD^b^<0·01%	Day 7 marrow blasts<25%
Good response	133	42
Bad response	68	40
N/A	6	17
**Long‐term outcome**	RFS^c^	CCR^d^ for at least 4 years
Relapsed	75	31
None	130	28
Censored	2	40
Median (Years)	6.5	N/A
Range (Years)	0.1–15.7	N/A

^a^ NCBI Gene Expression Omnibus

^b^ minimal residual disease

^c^ relapse‐free survival

^d^ complete continuous remission.

### 2.2 Pre‐processing of the microarray and gene filtering

The pre‐processing of the microarrays was performed using packages CustomCDF (version 1·0·5),^12^ hgu133plus2hsentrezg (version 23·0·0)^12^ and gcrma (version 2·54·0)^13^ in R environment (version 3·5·2). Platforms of the microarrays of COG P9906 and COG 1961 were Affymetrix Human Genome U133 Plus 2·0 Array,^6^, ^9^ and we re‐analysed the raw signal with the probes sets definition “hgu133plus2hsentrezg” developed by BRAINARRAY Microarray Lab because this definition is consistent to the up‐to‐date Entrez gene data.^12^ In the experimental CEL files, uninformative probe pairs, which gave mismatch signal intenser than perfect match signals in more than 90% of the samples, were removed. Probe set definition “hgu133plus2hsentrezg” was tailored according to the removal of the uninformative probe pairs. Probe sets were mapped to Entrez gene symbols and the expression levels of genes were calculated with the package gcrma.

Genes for the two cohorts were filtered using genefilter (version 1·64·0)^14^ in R environment. A gene was kept if its expression exceeded background threshold (expression value>100) in more than 10% samples and if the coefficient of variation (COV) of expression was greater than 1·0. The numbers of genes passing the filter were 527 and 959 for COG P9906 and COG 1961 respectively. We used the intersection of the two gene sets in the subsequent analysis, which contained 370 genes.

### 2.3 Non‐negative matrix factorization (NMF)

In the training set from COG P9906, the gene expression profiles consisted of *m* = 370 genes in *n* = 207 samples. We performed NMF to subgroup the samples, using package NMF (version 0·21·0)^15^ in R environment, which finds an approximation

V≈W·H

where *V* is the *m* × *n* matrix of the gene expression profiles of the training set, the so‐called basis components *W* is an *m* × *r* non‐negative matrix and the so‐called coefficients *H* is an *r* × *n* non‐negative matrix. A critical parameter in NMF is the factorization rank *r*, which defines the number of ALL subgroups in this study. Because there is yet no NMF algorithm finding the optimal approximation, the standard routine of NMF performs 30 runs with stochastic seeding and reports the best result which achieves the lowest approximation error. By comparing the NMF of ranks 2 to 12, we found out the best rank *r* = 4 using the criterion of minimum description length implemented by Squires *et al*.^16^ We confirmed the best rank by the consensus matrix method,^17^ which visualizes the subgrouping consensus among 30 runs for a rank.

Because the same *W* matrix was used in NMF of the training set and in reconstruction of NMF in the test set, in order to reduce the stochasticity and improve the reproducibility of subgrouping, we performed 60 runs for rank 4 NMF and constructed the *W* and *H* matrices by averaging among the top 20 runs with the lowest approximation error. The *H* matrix encoded the subgroups of samples: Sample *j* belonged to subgroup *i* if element *h_i_*_,_ *_j_* is the largest coefficients in column vector *h_j_*.

### 2.4 Logistic regression and Cox regression with elastic net regularization

Elastic net regularization is a combination of LASSO and ridge regularization. We performed the logistic regression and Cox regression with elastic net regularization using package glmnet (version 2·0–16)^18^, ^19^ in R environment. NMF split the training set into subgroups I, II, III and IV. Within each subgroup, a logistic regression model predicting MRD and a Cox regression model predicting RFS were built with parameter *α*=0·8, which assigned the mixing of LASSO and ridge regularization. Parameter *λ* controls the strength of the regularization that makes elastic net regression prefer simple models. As a result of a 10‐fold cross‐validation evaluated a series of models for different *λ*, a curve of the binomial deviance (for logistic regression) or the partial likelihood deviance (for Cox regression) in the cross‐validation indicates two values: *λ*_min_, at which the curve reached the minimum, and *λ*_1se_, at which the error was within 1 standard error of the minimum (Figure S1 and S2). The model given by *λ*_min_ has more coefficients and is usually more accuracy for the training set, while the model given by *λ*_1se_ is more robust and performs better in generalization. For predicting MRD, we extracted the model given by *λ*_1se_ (subgroups I and IV), and when the model given by *λ*_1se_ degenerated (all coefficients zero), we extracted the model given by *λ*_min_ (subgroups II and III). For predicting RFS, we extracted the simplest non‐degenerated model, which kept only one or two coefficients, because most of the models given by *λ*_min_ and *λ*_1se_ were degenerated.

For every subgroup, performance of a logistic regression model in the training set was evaluated by a receiver operating characteristic (ROC) curve and the area under the ROC curve (AUC). Performance of a Cox regression model in the training set was evaluated by concordance statistic (*c*)^20^ and inverse probability of censoring weighting (IPCW) estimation of cumulative time‐dependent ROC curve.^21^

### 2.5 Testing the regression models

We tested the subgrouping method and regression models with the data from COG 1961. NMF for the gene expression profile of the test set was reconstructed as:

$$H^{\prime }=W^{+}\cdot V^{\prime },$$

where *V′* was the matrix of the gene expression profiles of the test set, *W* was the basis component matrix which had been learned by NMF of the training set, and *H′* was the coefficient matrix yet to be determined. We solved *H′* as

$$H^{\prime }=W^{+}\cdot V^{\prime },$$

where *W* ^+^ is the Moore‐Penrose pseudoinverse of *W*:

$$W^{+}=T\cdot D^{‐1}S^{T},$$

according to singular value decomposition of *W* = *S D T* ^T^, where *S* and *T* are orthogonal, *T* ^T^ means *T* transposed and *D* is a diagonal matrix with the singular values. We subgrouped samples by finding for sample *j* the index of subgroup *i* which had the largest coefficient *h'_i_*_,_ *_j_* in column vector *h'_j_*.

MRD positivity of each patient was predicted by a logistic regression model learned by the same subgroup of the training set. Although MRD positivity of COG 1961 was not published, a patient was described as rapid early responder (RER) or slow early responder (SER), which also indicated early response to therapy measured by the percentage of blast cells in bone marrow. Early response is comparable to MRD‐based stratification, and RER signals a good outcome.^22^ Thus, we evaluated the performance of the logistic regression models using AUC comparing predicted MRD with early response.

RFS of each patient was predicted by a Cox regression model learned by the same subgroup of the training set. RFS of COG 1961 was not published, but a patient was described as in complete continuous remission (CCR) for more than 4 years or relapsed within 3 years of initial diagnosis. Thus, we ran Wilcoxon test to see whether predicted relapse risk of the patients reported to be CCR was significantly smaller than that of the relapsed patients.

## 3 RESULTS

### 3.1 Subgrouping by NMF

**FIGURE 2:**
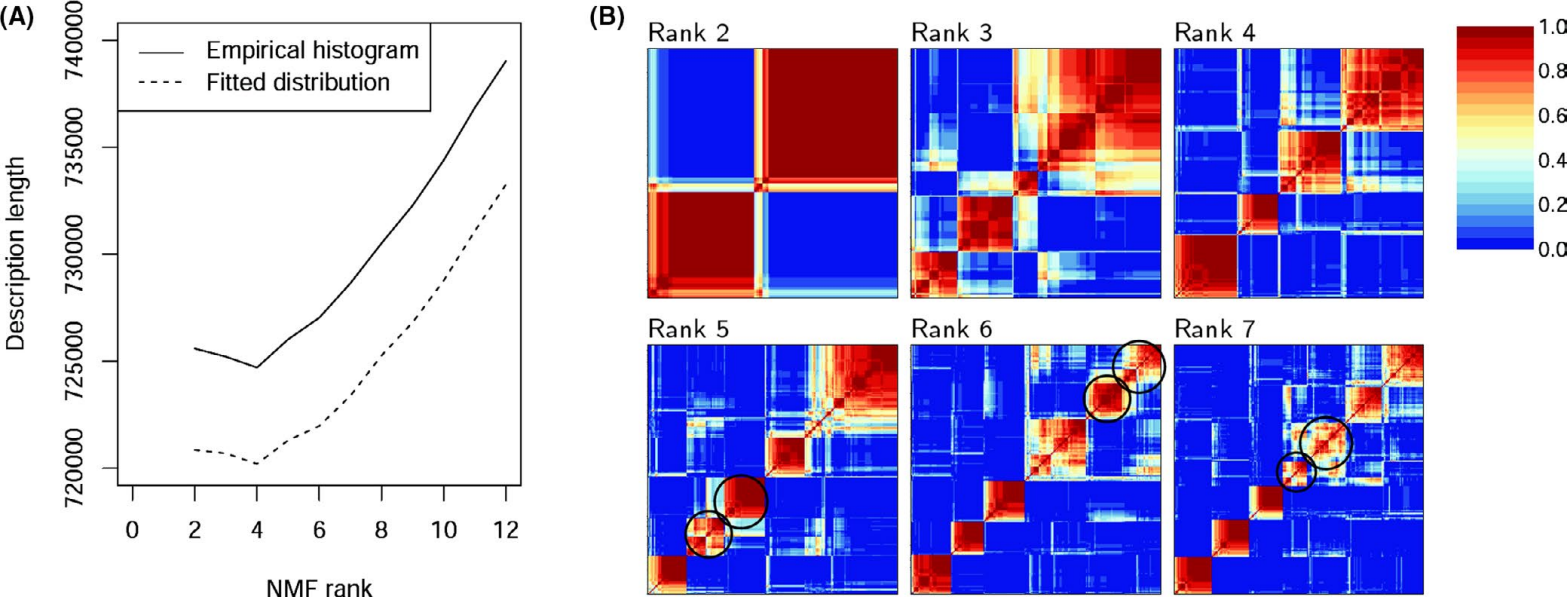
Comparison of NMF of different ranks shows that the training set constitutes of four subgroups. (A) The description length of the gene expression profiles of NMF of different ranks, calculated by methods based on empirical histogram and based on fitted distribution. (B) Consensus matrices for ranks 2 to 7, averaging over 30 runs. A pixel is coloured from blue, when the pair of samples are never in the same cluster, to red, when the pair of samples are always in the same cluster. In consensus matrices for ranks 5 to 7, circles indicate the blocks getting merged

In the training set, a meaningful rank of NMF should be much smaller than any of the two dimensions of the gene expression profiles (*m* = 370 and *n* = 207, Table S1). We ran NMF of ranks 2 to 12, and the minimum description length was minimized at rank 4 (Figure 2A), indicating that the information in the training set was effectively distilled by rank 4 NMF. Visualization of the quality and stability of NMF by the consensus matrix method^17^ shows that rank 4 is better than others (Figure 2B). For the rank 4 NMF, large values of consensus among 30 runs are collected in four square blocks along the diagonal. For the rank 2 and 3 NMF, the square block pattern gets blurred, that is, the blocks have nested structure in which a few dispersed cases near the boundary between blocks get associated with other subgroups. When the rank is larger than 4, the association of dangling cases in different subgroups merges square blocks, reducing the overall number of independent blocks. For example, only four square blocks are clearly displayed in the consensus matrix for rank 5 NMF, where two blocks merge (Figure 2B, circles in the matrix for rank 5).

**FIGURE 3:**
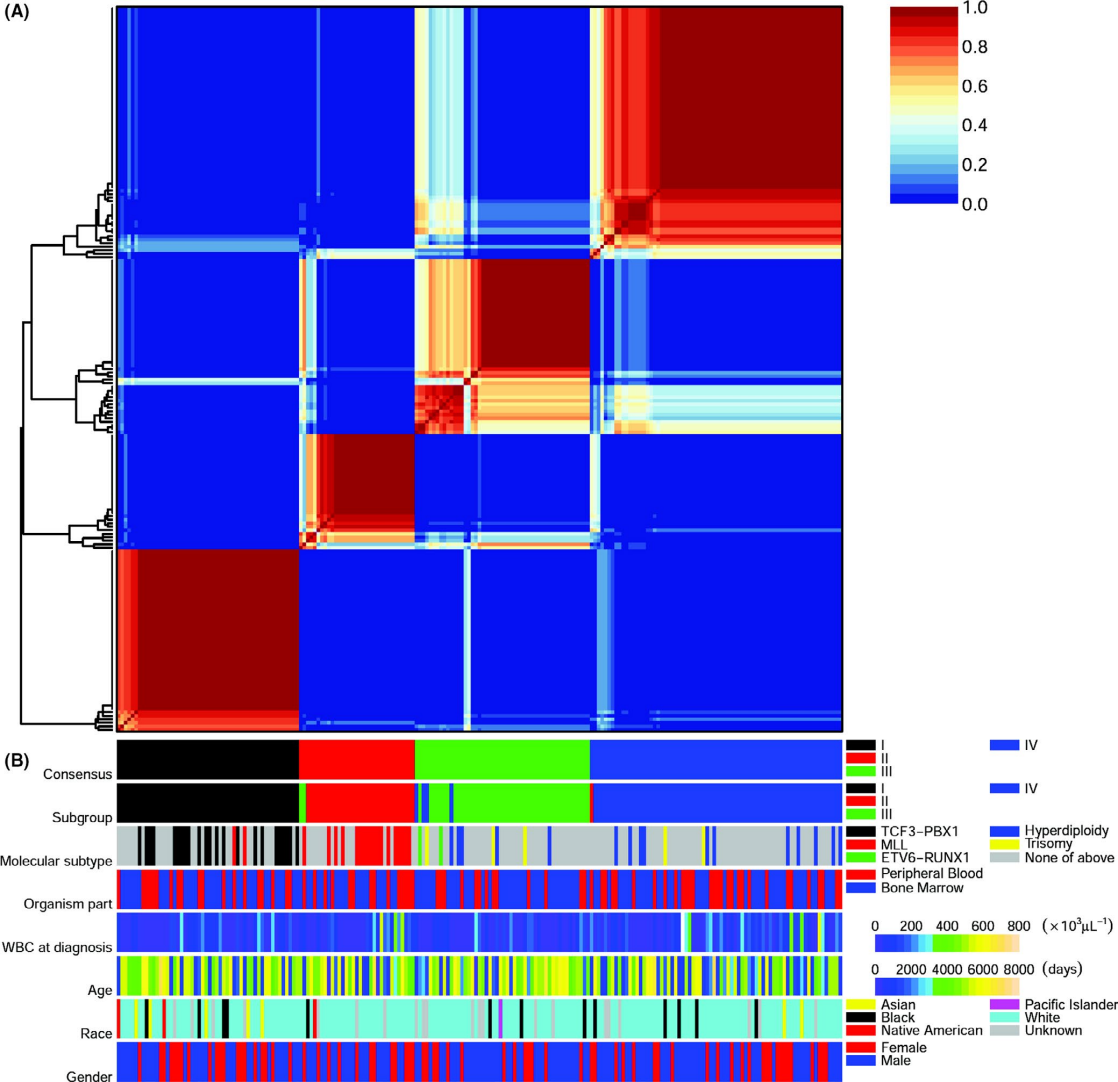
Subgrouping of the training set by NMF and the association of subgroups with genetic subtypes. (A) A consensus matrix for rank 4 NMF, averaging the top 20 from 60 runs. A pixel is coloured from blue, when the pair of samples are never in the same cluster, to red, when the pair of samples are always in the same cluster. On the left of the consensus matrix, the hierarchical clustering of samples is shown. (B) Annotation tracks for consensus, subgroups, genetic subtypes, organism parts, white blood cell at diagnosis, age, race and gender. Legends and scales of the tracks are shown on the right, where “Hyperdiploidy” is short for “Hyperdiploidy without trisomy of both chromosomes 4 and 10”, and “Trisomy of 4/10” is short for “Trisomy of both chromosomes 4 and 10”

For subgrouping by rank 4 NMF, we constructed the ***W*** (Data S1) and ***H*** matrices by taking the average among the top 20 runs with the lowest approximation error. The consensus matrix for the top 20 runs clearly displays a four‐square‐block pattern (Figure 3A) cleaner than that for 30 runs (Figure 2B, rank 4). The subgrouping (Figure 3B, the line “Subgroup”) was almost identical to the grouping suggested by the consensus matrix (Figure 3B, the line “Consensus”), except a few cases. These exceptions also played the dangling cases in the consensus matrix (Figure 3A).

Subgroups I, II, III and IV contained 52, 31, 52 and 72 samples respectively. Although NMF performed unsupervised clustering, the subgroups were roughly overlapped with the genetic subtypes of paediatric B‐precursor ALL published by COG P9906 (Figure 3B, the line “Subtype”). In subgroup I, there were 23 patients with subtype TCF3‐PBX1, 2 with subtype MLL and 27 with undefined subtypes. Subgroup II consisted of 17 patients with subtype MLL and 14 with undefined subtypes. Subgroups III and IV were more heterogeneous than the previous two subgroups. Subgroup III consisted of 2 patients with subtype ETV6‐RUNX1, 1 with subtype MLL, 5 with hyperdiploidy without trisomy of both chromosomes 4 and 10, 3 with trisomy of both chromosomes 4 and 10, and 41 with undefined subtypes. Subgroup IV consisted of 11 patients with hyperdiploidy without trisomy of both chromosomes 4 and 10, 2 with trisomy of both chromosomes 4 and 10, and 59 with undefined subtypes. Beside overlapped with genetic subtypes, the subgroups displayed little association with organism parts of the samples, white blood counts at diagnosis and personal features like gender, race and ethnicity (Figure 3B).

Few associations between outcomes and subgrouping were detected (Figure S3). Subgroups I and IV were different in the early response to therapy, which was MRD in the training set (Figure S3A) and was rapid versus slow early response in the test set (Figure S3C). Although in RFS in the training set, differences were detected between subgroups I and IV and between subgroups III and IV (Figure S3B), we found no difference of distribution of CCR and relapse cases between subgroups in the test set (Figure S3D).

### 3.2 Logistic regression models predicting MRD

**FIGURE 4:**
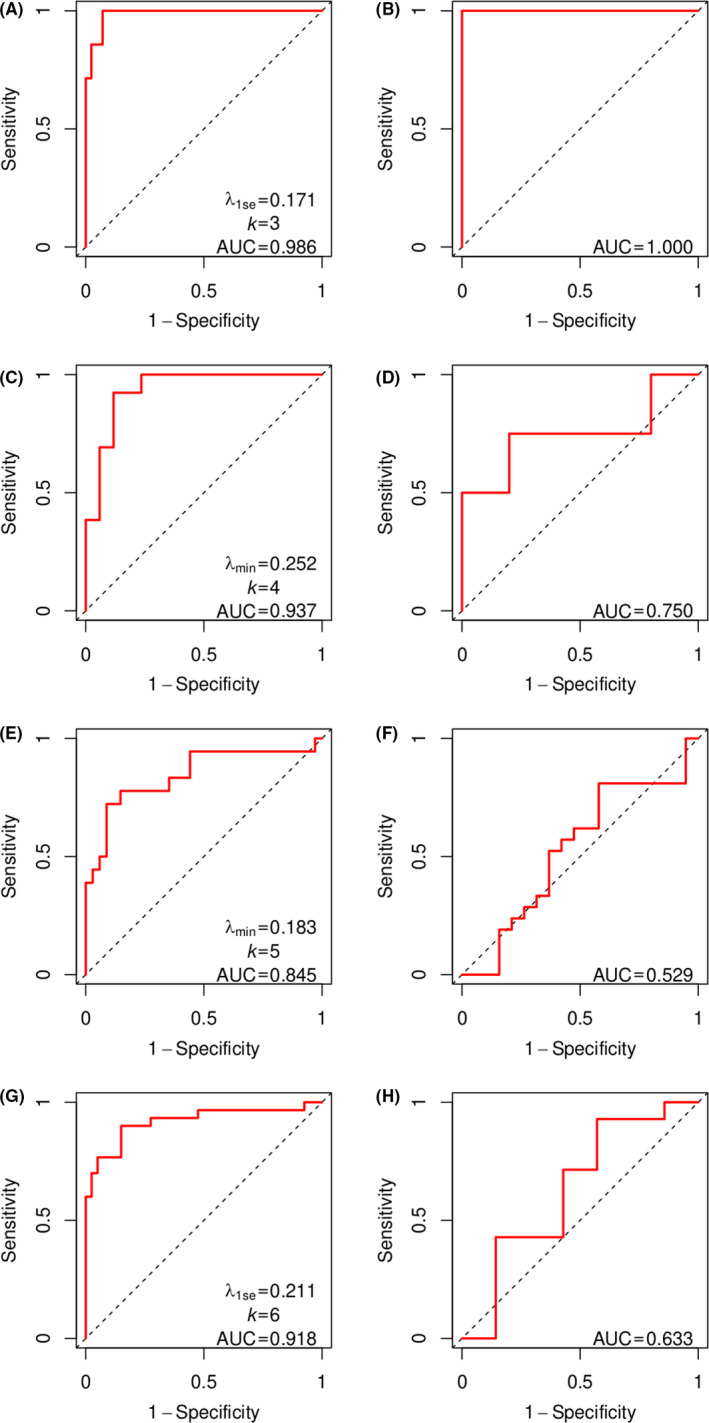
Performance of logistic regression models predicting MRD. (A, C, E and G) ROC curves (red solid lines) for subgroups I, II, III and IV in the training set. The value of *λ*, the number of coefficients (*k*) and the area under the ROC curve (AUC) are shown for each subgroup. The diagonal dashed line is the no discrimination line. (B, D, F and H) ROC curves (red solid lines) comparing predicted MRD with early response for subgroups I, II, III and IV in the test set. The AUC is shown for each subgroup

Elastic net regression produced a series of logistic regression models controlled by the parameter *λ*. According to 10‐fold cross‐validations, we extracted the model of *λ*_1se_=0·171 for subgroup I (Figure S1A, Table S2). The model gave an impressed performance in the training set (AUC = 0·986, Figure 4A). In the test set, the model even gave a perfect performance (AUC = 1·0, Figure 4B).

For subgroup II, the model of *λ*_1se_ had all coefficients zero (Figure S1B). Such a degenerated model performs poorly for subgroup II, since MRD in this subgroup is not uniformly the same status: There are 17 cases of negative MRD and 13 cases of positive (and 1 case with unknown status). We extracted the model of *λ*_min_ = 0·252 (Table S2), which performed well in the training set (AUC = 0·937, Figure 4C). In the test set, the model gave an acceptable performance (AUC = 0·750, Figure 4D).

For subgroup III, the model of *λ*_1se_ was also degenerated (Figure S1C), and we extracted the model of *λ*_min_ = 0·183 (Table S2), which performed well in the training set (AUC = 0·845, Figure 4E). In the test set, the models performed poorly (AUC = 0·529, Figure 4F). For subgroup IV, the model of *λ*_1se_ = 0·211 (Figure S1D and Table S2) performed well in the training set (AUC = 0·918, Figure 4G) but became much worse in the test set (AUC = 0·633, Figure 4H).

### 3.3 Cox regression models predicting RFS

**FIGURE 5:**
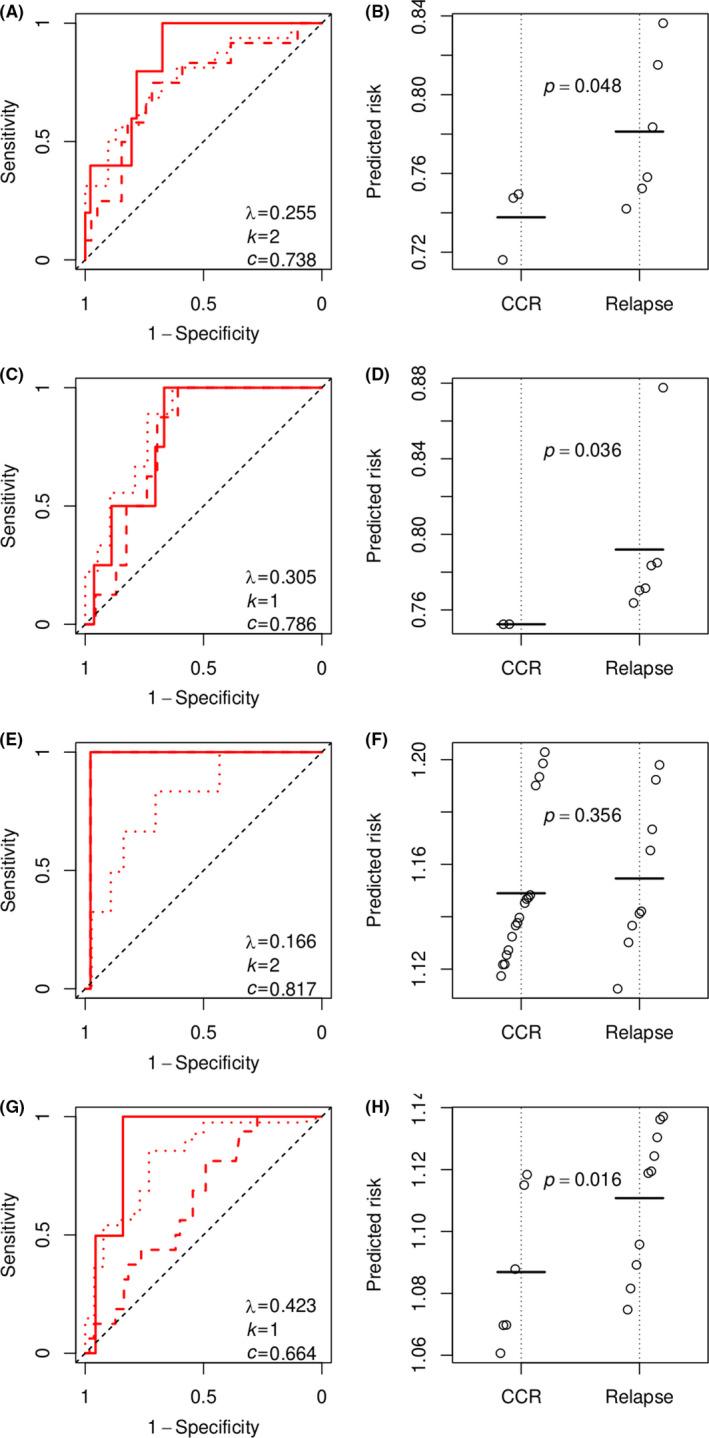
Performance of the Cox regression models predicting RFS. (A, C, E and G) Time‐dependent ROC curves for subgroups I, II, III and IV in the training set. Solid, dashed and dotted red lines indicate 1‐year, 2‐year and 5‐year RFS respectively. The value of *λ*, the number of coefficients (*k*) and concordance statistic (*c*) of the Cox regression model are shown for each subgroup. The diagonal dashed line is the no discrimination line. (B, D, F and H) Wilcoxon tests of the Cox regression models in predicting relapse within 3 years for subgroups I, II, III and IV in the test set. Points indicate the risk predicted by the Cox regression model. Horizontal bars indicate the averages among CCR patients and among relapsed patients. The *p* value of a Wilcoxon test is shown for every subgroup

We used Cox regression models to study the relationship between gene expression profiles and RFS. The cross‐validation identified models of *λ*_min_ for subgroups I and IV only, but models of *λ*_min_ for subgroups II and III and models of *λ*_1se_ for all subgroups were degenerated (Figure S2). Although the model of *λ*_min_ for subgroup I described RFS perfectly (Figure S4A, *c* = 0·946), the large number (*k* = 22) of coefficients may affect its generalization to the test set (Figure S4B). The model of *λ*_min_ for subgroup IV performed (Figure S4C, *c* = 0·669; and Figure S4D) similarly to that of the simplest non‐degenerated model with only one coefficient (Figure 5G, c = 0·664; and Figure 5H). For consistency, we extracted the simplest non‐degenerated models for all subgroups. These models had only one or two coefficients (Figure 5A,C,E and G, and Table S2). In the training set, the concordance statistic of the models with RFS in four subgroups ranged from 0·664 to 0·817 (Figure S5). Performance in the test set was evaluated by Wilcoxon tests, showing whether the risk of the relapsed patients was significantly larger than that of patients reported to be CCR. Wilcoxon tests in subgroups I, II and IV yielded statistically significance results (*p* = 0·048, 0·036 and 0·016, Figure 5,B,D and H), suggesting the three models were well generalized to the test set. The model for subgroup III, however, failed to discriminate the CCR and relapsed patients (*p* = 0·356, Figure 5F).

Displayed by time‐dependent ROC curves, the models for subgroups I and II performed similar in predicting the 1‐year, 2‐year and 5‐year RFS (Figure 5A and C). The model for subgroup III, however, lost a little prediction power for 5‐year RFS (Figure 5E), the AUC of which was 0·978 for 1‐year and 2‐year RFS and drops to 0·801 for 5‐year RFS. The model for subgroup IV performed well for 1‐year RFS (AUC = 0·891), lost most prediction power for 2‐year RFS (AUC = 0·654) and restored the prediction power for 5‐year prediction (AUC = 0·828, Figure 5G).

### 3.4 Genes associated with the outcomes in paediatric ALL

Genes providing coefficients in the regression models are important biomarkers for predicting outcomes in paediatric B‐precursor ALL. Most of the significant genes are unique to specific subgroups and outcomes, except gene *npdc1*, which is shared by the model predicting MRD for subgroup III and the models predicting MRD and RFS for subgroup IV (Table S2).

In subgroup I, the model predicting MRD highlights the positive correlation of expression of *cd34*, *dipk1c* (also known as *fam69c* and *c18orf51*) and *mrc1* (also known as *cd206*) with MRD positivity or SER. Gene *cd34* encodes protein that may participate in the attachment of stem cells to the bone marrow extracellular matrix or to stromal cells, and has been associated to outcomes of acute myeloid leukaemia (AML),^23^, ^24^ but its relevance to ALL, especially to the subtype TCF3‐PBX1, remains controversial.^25^, ^26^ Gene *mrc1 *has been reported to be expressed in a group of peritoneal leukaemia‐associated macrophages.^27^ The performance of the model in the training set and the test set suggests that these genes are fundamental or at least highly correlated with fundamental pathways in the treatment and early response in subgroup I or subtype TCF3‐PBX1. The model predicting RFS for subgroup I suggests genes *fam241a* and *mgme1* are protective factors for the patients. *mgme1* encodes a protein maintaining mitochondrial genome synthesis.^28^

In subgroup IV, the model predicting MRD has coefficients of genes *npdc1*, *cd38*, *kcnk12*, *prx*, *smad1* and *ptger2*, all of which but *cd38* are risk factors; the model predicting RFS has only one coefficient of gene *npdc1*. *npdc1* plays a risk factor in both models and also in the model predicting MRD for subgroup III. This gene has been reported to be significantly up‐regulated at relapse of AML and has been used in a highly prognostic signature in AML,^29^, ^30^ suggesting it is fundamental in leukaemia progression.

In subgroup II, the model predicting MRD reveals *bre*‐*as1* (also known as *babam2*‐*as1*) and *anxa1* as risk factor and *ddit4 l* and *wwc3* as protective factor. Dysregulations of *anxa1* have been detected in multiple tumours.^31^, ^32^ Gene *wwc3 *has been linked to Hippo signalling cascade related to tumorigenesis.^33^, ^34^ The model predicting RFS suggests gene *cerk* is a protective factors for the patients. Gene *cerk* regulates the migration of bone marrow‐derived mesenchymal stem cells.^35^ In subgroup III, although both models predicting MRD and RFS perform poorly, the model predicting MRD identifies *parp15*, *prxl2c*, *npdc1*, *clec14a* and *opn3*, and the model predicting RFS identifies *c1qtnf4* (also known as *ctrp4*) and *scml1*, all of which but *parp15* and *opn3* are risk factor. Expression level of *opn3 *has been negatively correlated with the activity of anti‐apoptotic pathway in hepatocellular carcinoma.^36^ Gene *c1qtnf4* can promote tumour cell survival and tumour resistance against apoptosis induced by chemotherapeutics.^37^

## 4 DISCUSSION

Paediatric ALL consists of various subtypes. Prognostic signatures may exist within biologic subtypes of ALL only.^6^ NMF has been applied to leukaemia gene expression profiles previously, successfully recognizing the classes of AML, T‐ALL and B‐ALL.^17^ In our study, NMF is applied to subgrouping of a cohort of high‐risk B‐precursor ALL. The subgroups roughly overlap with subtypes determined by genetic abnormalities. The coincidence of NMF subgroups and genetic subtypes may be a consequence of specific types of dysregulation of the gene network, for example, it has been reported that in subtype ETV6‐RUNX1, expression of multiple target genes is induced by the chimeric transcription factor ETV6‐RUNX1.^38^ All patients with subtype TCF3‐PBX1 were classified as subgroup I. Patients with subtype MLL were mainly classified as subgroup II, except a few classified as subgroups I and III. The models predicting MRD and RFS in subgroup I performed perfectly in the training set and was generalized well to the test set. Roughly speaking, subtype TCF3‐PBX1 has a clear and unique gene expression pattern that can be recognized by NMF in such a sample size. Subtype MLL seems heterogeneous, among which some cases had gene expression patterns similar to subtype TCF3‐PBX1 and were classified as subgroup I. Performance of the models predicting MRD and RFS in subgroup II was not as good as in subgroup I but still acceptable. Patients with hyperdiploidy without trisomy of both chromosomes 4 and 10 and patients with trisomy of both chromosomes 4 and 10 were dispersed in subgroups III and IV. The gene expression patterns of the two subgroups may not be clearly depicted with such a sample size. The poor performance in generalization of the models predicting MRD and RFS to the test set in the two subgroups III and IV also suggests the complexity of the two subgroups and these subtypes. Patients with subtype ETV6‐RUNX1, the only two patients with such a low‐risk subtype included in the cohort of COG P9906, were classified as subgroup III. There were many cases without known subtypes and were subgrouped together with known subtypes, suggesting the similarity in gene expression patterns. Although gene expression profiles provide many features to define subtypes, the sample size limited the number of subgroups that NMF is able to identify. The subgrouping found a good balance between over‐fitting with many minor subtypes and a single global signature. The prediction performance of the model predicting MRD for subgroup I is much better than a global model published previously (AUC = 0·8),^6^, ^9^ suggesting such a divide‐and‐conquer strategy effectively picks out subgroup I whose pattern is clear given the available samples.

We trained the model with the cohort of COG P9906, and tested the models with the cohort of COG 1961. The early prediction of MRD is clinical relevant for adjustment of therapy. COG 1961 reported the early response instead of MRD, and we used it to evaluate our prediction. Although the early response was determined on day 7 and the measurement was different from that of MRD, the consistency of the two data sets has been verified by several studies.^9^, ^39^, ^40^ For the test set, the models for subgroups II, III and IV performed worse than for subgroup I. The worse performance may stem from the minor difference between the designs of the two clinical trials, and, more importantly, the heterogeneity of the genetic subtyping of both data sets. COG P9906 mainly recruited high‐risk B‐precursor ALL patients, while patients in COG 1961 totally lacked known genetic subtypes. We subgrouped the patients in COG 1961 by the criterion learned from the gene expression profiles of COG P9906. Performance of the subgrouping cannot be evaluated with the test set. However, the performance of the MRD models in the test set meets our expectation that logistic regression performs well in subgroup I, moderate in subgroups II and IV and poor in subgroup III because subgroups II, III and IV are heterogeneous and the models may suffer from the small sample size after subgrouping. Actually, the models for subgroups II, III and IV performs badly only in the test set but very well in the training set, suggesting the problem of small sample size and over‐fitting.

Comparison between the models predicting MRD and RFS regarding their performance suggests that the models predicting MRD are better in generalization, that is, all models predicting RFS, except the model for subgroups III, predict risk score discriminating the CCR and relapsed patients. The models predicting RFS involve different genes from the models predicting MRD, except in subgroup IV. Since expression profiles of different genes may be highly correlated, the different genes may simply result from random selection by the regression with elastic net regularization. It is also possible that early response and long‐term effect need to be evaluated with different genes in some subtypes, which deserved further studies because it has been reported that relapses occur in some paediatric ALL patients with an excellent (negative) MRD.^9^, ^41^

The combination method of NMF and elastic net regression implemented in this study subgroups patients with B‐precursor ALL and predicts the outcomes. Improvement of the prediction may be attributable to the handling of heterogeneity of B‐precursor ALL.

## CONFLICT OF INTERESTS

The authors declare that they have no conflict of interest.

## Supporting information

Appendix S1

Data S1

## Data Availability

Supplementary information, including figures and tables, is available at the journal's website. The data used for this analysis are available at https://portal.gdc.cancer.gov/projects and the NCBI Gene Expression Omnibus database.
