# Supplementary material for: Subgrouping by gene expression profiles to improve relapse risk prediction in paediatric B‐precursor acute lymphoblastic leukaemia
Source: Cancer Med. 2021 May 13;10(11):3782–93. doi: 10.1002/cam4.3842 (PMC8178509; doi:10.1002/cam4.3842)
Supplement: Supplementary file 1 — Appendix S1 [file CAM4-10-3782-s001.docx]

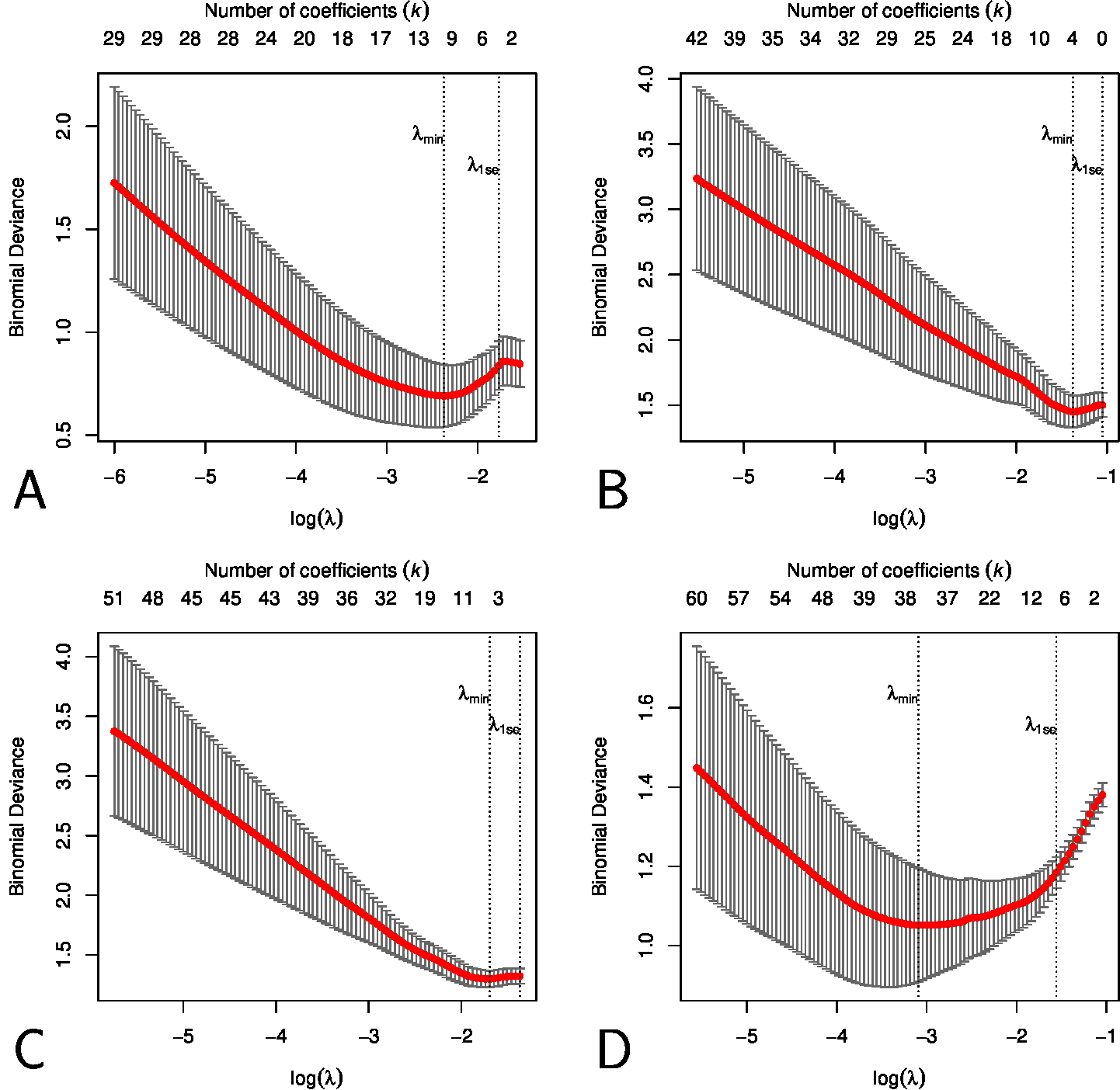


**Figure S1: Ten-fold cross-validation curves of the logistic regression with elastic net regularization.** **(A, B, C, and D)** Curves for subgroups I, II, III, and IV. The red points indicate binomial deviance in the cross-validation, with black error bars for standard deviation. Vertical dotted lines indicated *λ*_min_, at which the curve reached the minimum, and *λ*_1se_, at which the error was within 1 standard error of the minimum. The axis above indicates the number of coefficients (*k*).


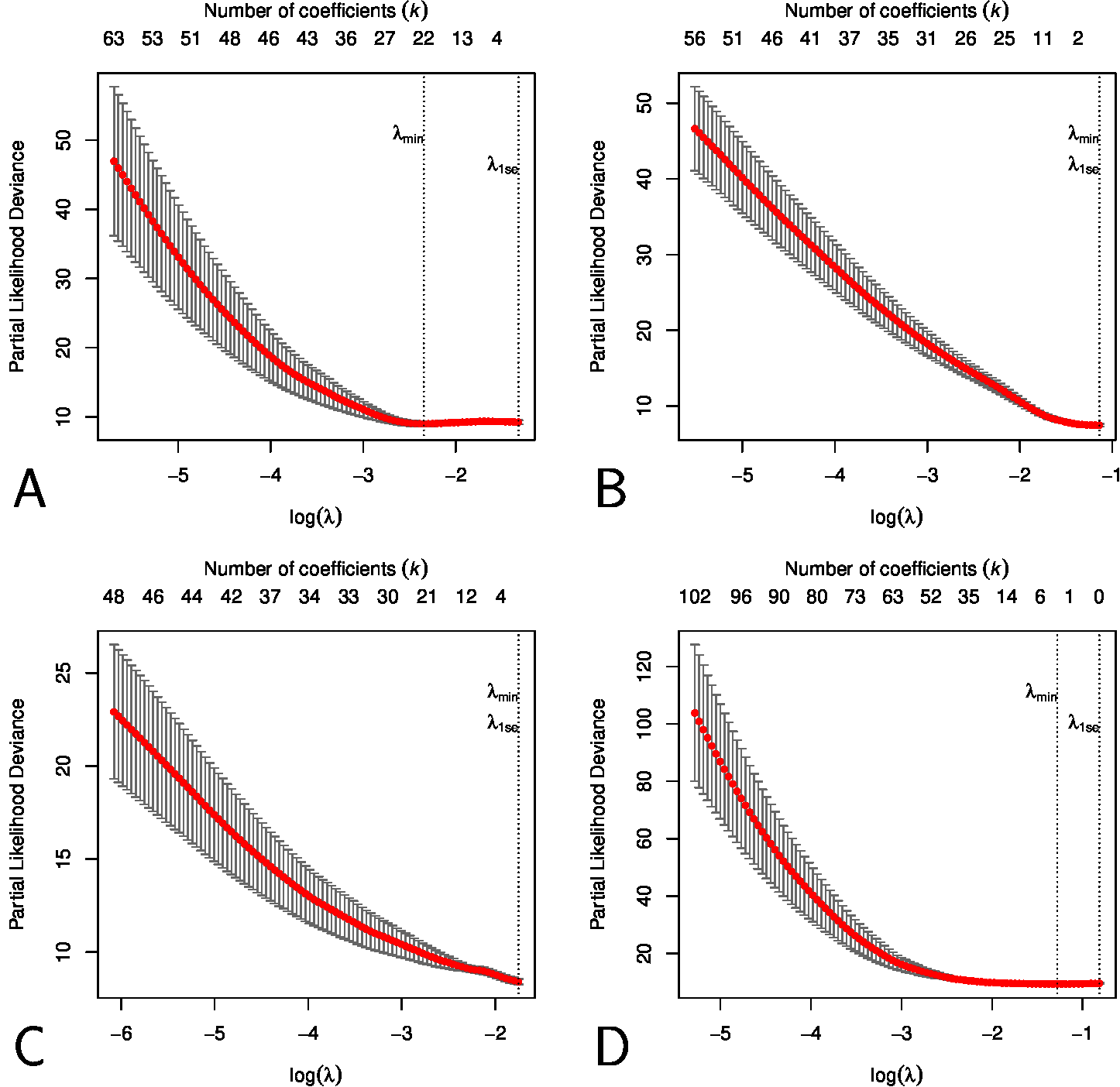


**Figure S2: Ten-fold cross-validation curves of the Cox regression with elastic net regularization.** **(A, B, C, and D)** Curves for subgroups I, II, III, and IV. The red points indicate deviance in the cross-validation, with black error bars for standard deviation. Vertical dotted lines indicated *λ*_min_, at which the curve reached the minimum, and *λ*_1se_, at which the error was within 1 standard error of the minimum. The axis above indicates the number of coefficients (*k*).


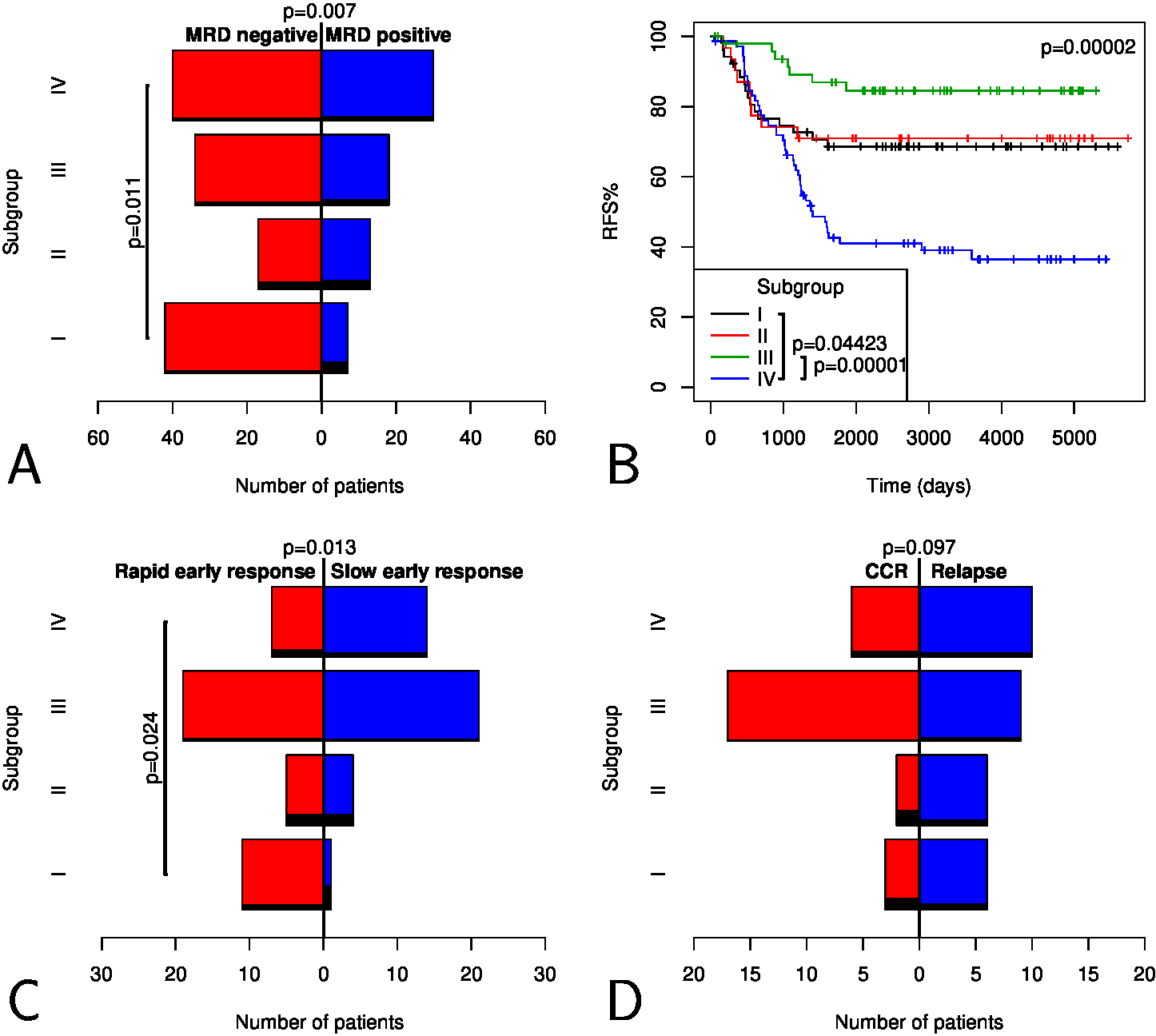


**Figure S3: Association of outcomes with subgroups in the training set and the test set. (A)** Distribution of MRD negative and positive cases in training set. **(B)** Kaplan-Meier survival curves of RFS in training set. **(C)** Distribution of rapid and slow early response cases in test set. **(D)** Distribution of continuous complete remission (CCR) and relapse cases in the test set. On top of the plot (A, C, and D), a *p* value of a χ^2^ test shows whether there is a difference between the expected frequencies and the observed frequencies in one or more categories of a contingency table. In the top right corner of the plot (B), a *p* value shows whether there is a difference between two or more survival curves. The differences between subgroups are also indicated if significant (*p*<0.05).


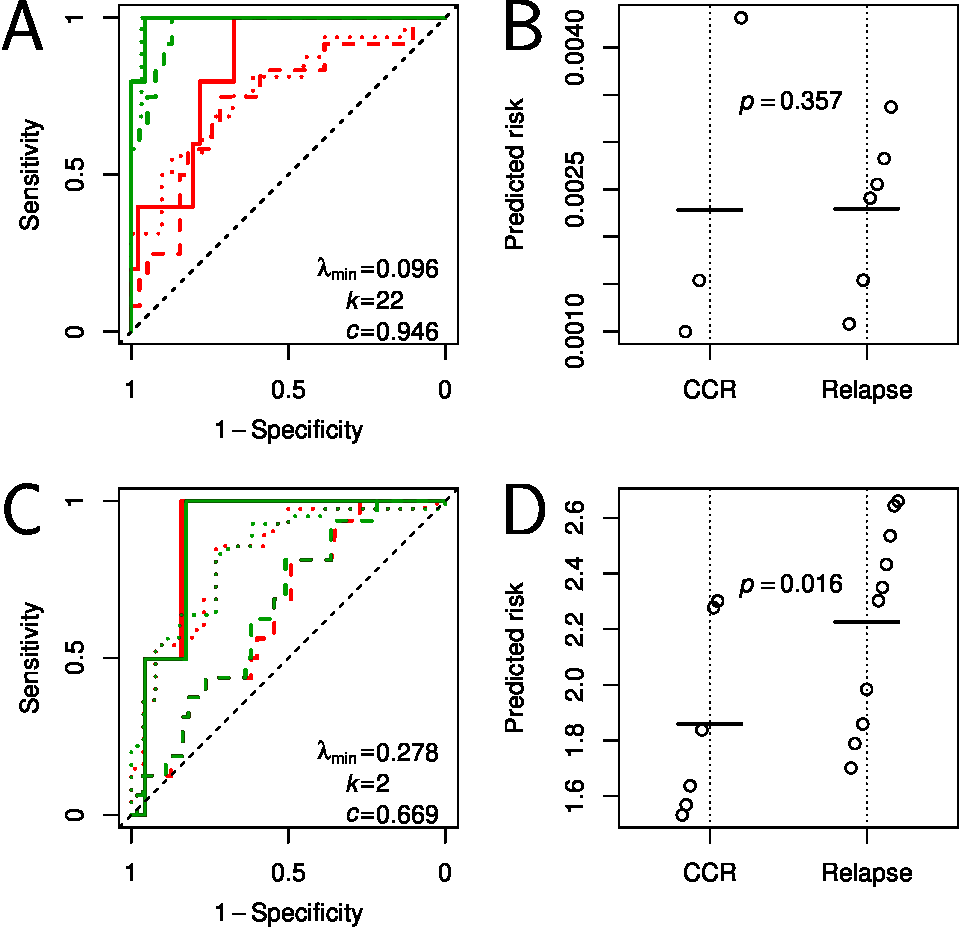


**Figure S4 Performance of the Cox regression models predicting RFS.** **(A and C)** Time-dependent ROC curves for subgroups I and IV in training set. Solid, dashed, and dotted lines indicate 1-year, 2-year, and 5-year RFS, respectively. Red lines are for the simplest non-degenerated model (also shown in Figure 4, A and G), which kept only one or two coefficients, while green lines are for the model given by *λ*_min_. The value of *λ*, the number of coefficients (*k*), and concordance (*c*) of the Cox regression model of *λ*_min_ are shown for each subgroup. The diagonal dashed line is the no discrimination line. **(B and D)** Wilcoxon tests of the Cox regression models of *λ*_min_ in predicting relapsed within 3 years for subgroups I and IV in the test set. Points indicate the risk predicted by the Cox regression model. Horizontal bars indicate the averages among CCR patients and among relapsed patients. The *p* value of Wilcoxon test is shown for every subgroup.


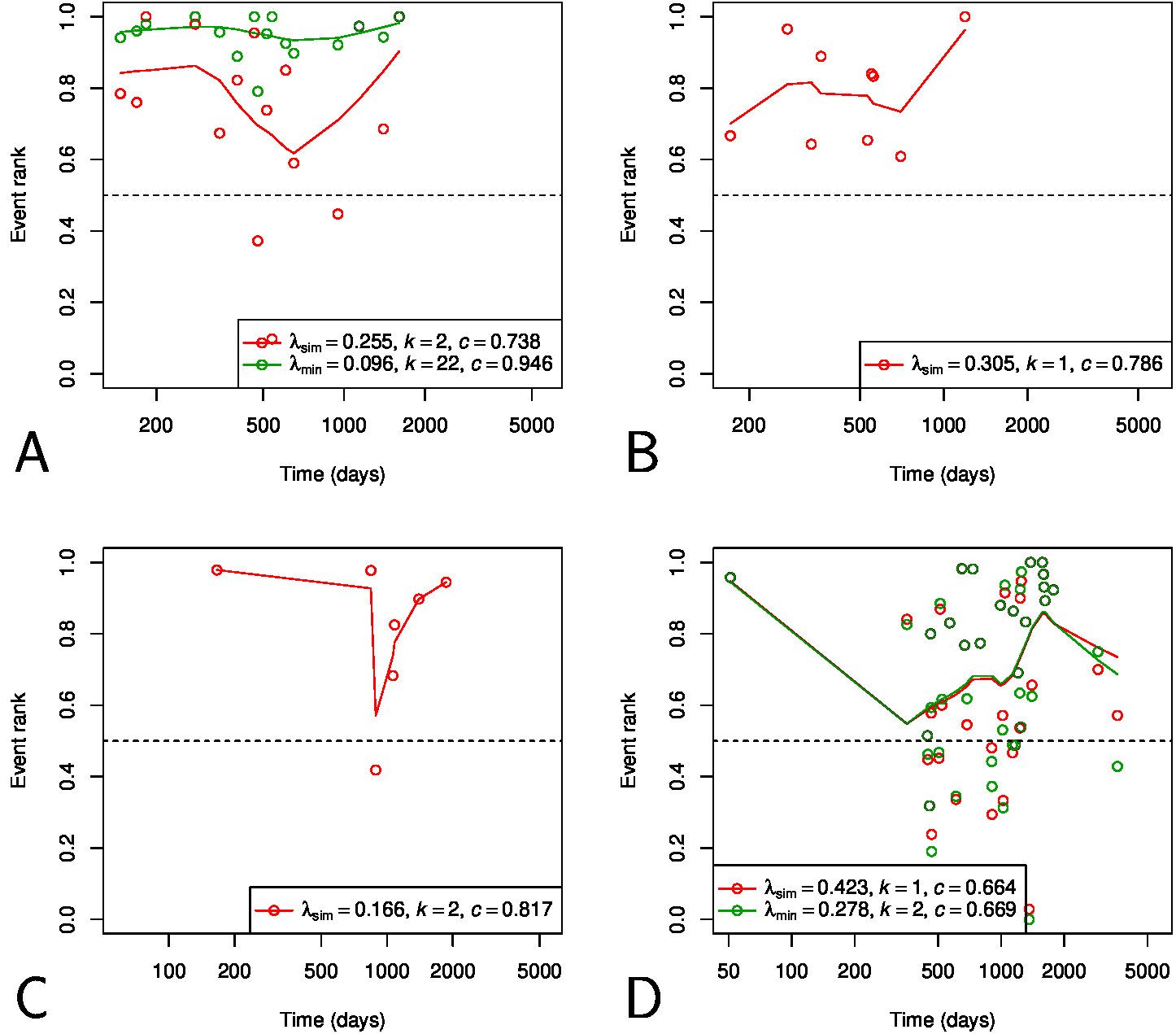


**Figure S5: Concordance of each relapse time and its predicted risk score. (A, B, C, and D)** Concordance for models in subgroups I, II, III, and IV. The vertical axis denotes the rank of the predicted risk score for a patient who was relapsed, where 1 means the patient had the highest risk score at the relapse and 0 means the lowest. Red lines are for the simplest non-degenerated model (*λ*_sim_), while green lines are for the model given by *λ*_min_. The value of *λ*, the number of coefficients (*k*), and concordance (*c*) are shown for each Cox regression model. The horizontal dashed line denotes a trivial rank (0.5), events below which have little predictive ability.

**Table S1 Genes (HGNC symbols) used in NMF**

| ABCA1 | CD34 | EFNA1 | HSPA1A | LPAR6 | NXN | S100A10 | SV2A |
| --- | --- | --- | --- | --- | --- | --- | --- |
| ABHD15 | CD38 | EGR1 | ID2 | LRMP | ODC1 | S100A16 | TCFL5 |
| ABHD17C | CD3D | EIF1AY | ID3 | LRRC70 | OPN3 | S100A8 | TCL1A |
| ACSL1 | CD44 | EIPR1 | IER3 | LSP1 | P2RX5 | S100A9 | TCL1B |
| ADM | CD72 | ELL2 | IFI30 | LST1 | P2RY14 | SCML1 | TENT5C |
| AEBP1 | CD99P1 | EMP1 | IFI44 | LTB | PAM | SCN3A | TERF2 |
| AGPS | CDC20 | F13A1 | IFI44L | LY9 | PARP15 | SEC11C | TESC |
| AHR | CDC42EP3 | FAM171A1 | IFIT3 | LYZ | PARP8 | SELL | TFDP1 |
| AHSP | CEBPB | FAM241A | IFITM1 | MACROD2 | PBX3 | SERINC2 | TIAM2 |
| ALKAL2 | CERK | FAM49A | IGF2BP3 | MAFF | PCDH9 | SESN1 | TIMP2 |
| ALOX5AP | CHST12 | FAM78A | IGFBP7 | MAN1A1 | PELI1 | SFXN1 | TMED6 |
| AMOT | CLEC11A | FAM83D | IGKC | MANF | PHACTR1 | SGK1 | TMSB15A |
| ANKRD33B | CLEC14A | FAT1 | IGLL1 | MAP3K5 | PHACTR3 | SH3BP5 | TNF |
| ANXA1 | CMPK2 | FKBP11 | IGLL3P | MEST | PHYH | SHANK3 | TNFRSF21 |
| ANXA2R | CMTM2 | FKBP2 | IL17RA | METRNL | PKIG | SHCBP1 | TNFSF13B |
| ANXA5 | CMTM8 | FKBP5 | IL1B | MGME1 | PLEK | SIDT1 | TNFSF4 |
| AREG | COMMD3 | FLT3 | IL7R | MGST3 | PLEKHG1 | SIMC1 | TOP2A |
| ARHGAP29 | CORO1C | FOXO6 | INAFM2 | MIR155HG | PLK2 | SIPA1L2 | TOR1B |
| ARHGEF10 | COTL1 | FUCA1 | IRX1 | MN1 | PON2 | SLC12A6 | TOX2 |
| ARL4C | CPXM1 | GADD45A | IRX2 | MOB1B | PRAG1 | SLC15A4 | TPBG |
| ARMCX1 | CRIP1 | GADD45B | ISG15 | MPO | PRDX1 | SLC25A39 | TRIM24 |
| ARRDC4 | CRLF2 | GFOD1 | ITGA6 | MRC1 | PRDX2 | SLC27A3 | TSPAN17 |
| ASAP2 | CSF2RB | GNG11 | ITGAM | MRPS31 | PRKAR2B | SLC2A3 | TUBB2A |
| ATP2B1-AS1 | CSRP2 | GOLGA8A | ITGB2 | MS4A1 | PRKCZ | SLC2A5 | TUBB6 |
| BACH2 | CST7 | GPM6B | ITM2A | MT2A | PRKX | SLC35E3 | TUSC1 |
| BASP1 | CTGF | GPR160 | JCHAIN | MTCL1 | PROM1 | SLC43A2 | TXLNGY |
| BCAT1 | CTHRC1 | GPR18 | KANK2 | MTHFD2 | PRX | SMAD1 | TYROBP |
| BEX4 | CXCL8 | GPR183 | KCNK12 | MTIF3 | PRXL2C | SMAD7 | UAP1 |
| BLK | CXorf21 | GUSBP11 | KCNK17 | MX1 | PTGER2 | SMAGP | UBXN10-AS1 |
| BLVRB | CXXC5 | GYG1 | KCTD12 | MX2 | PTPRK | SMAP2 | ULK1 |
| BMF | CYB5A | GYPC | KHDRBS3 | MYC | PVRIG | SNTG2-AS1 | UROD |
| BMP2 | CYB5R2 | GZMA | KLF9 | MYLK | QSOX2 | SNX9 | USP36 |
| BRE-AS1 | CYFIP1 | H1F0 | KLHL2 | NCF2 | RAB37 | SOCS5 | VAT1L |
| BTBD3 | CYTL1 | HACD1 | LAMP5 | NDUFA8 | RADIL | SOX11 | VPS26B |
| BTBD6 | DAD1 | HBD | LCK | NEURL1B | RAG1 | SPON2 | WASF1 |
| BTG3 | DDIT4L | HBG2 | LCN10 | NFIL3 | RASD1 | SPRY1 | WFS1 |
| C11orf96 | DEF8 | HBM | LGALS1 | NFKBID | RASGRP1 | SPRY2 | WWC3 |
| C12orf75 | DHRS3 | HCK | LGALS3 | NID2 | RCBTB2 | SPTA1 | XIST |
| C1QTNF4 | DIPK1C | HEXIM1 | LGALS3BP | NINJ1 | REM2 | SQOR | YES1 |
| CA1 | DPEP1 | HHIP-AS1 | LGR6 | NPDC1 | RGCC | SRGN | ZNF331 |
| CA2 | DSTN | HIST1H1C | LILRB1 | NPY | RGL1 | STAG3 | ZNF608 |
| CAPG | DTX1 | HIST1H2AM | LINC00865 | NR4A1 | RGS1 | STAP1 |  |
| CAPN2 | DTX4 | HIST1H2BD | LINC00926 | NR4A2 | RGS10 | STAT1 |  |
| CARD16 | DUSP2 | HIST1H2BG | LINC00960 | NRN1 | RGS2 | STIM2 |  |
| CAT | DYSF | HIST1H3D | LMO2 | NSMCE1 | RPPH1 | STK39 |  |
| CCDC81 | EAF2 | HLA-DQB1 | LOC105374869 | NT5E | RPS20P22 | STOM |  |
| CD27 | ECM1 | HMBS | LONRF1 | NUCB2 | RUBCNL | SUPT3H |  |

**Table S2 Coefficients of genes (HGNC symbols) used by the models**

|  | Logistic model predicting MRD | | | | | | Cox model predicting RFS | | | | | |
| --- | --- | --- | --- | --- | --- | --- | --- | --- | --- | --- | --- | --- |
| Subgroup | I | | II | III | IV | | I | | II | III | IV | |
| Extracted Model | *λ*_min_= 0.093 | *λ*_1se_= 0.171 | *λ*_min_= 0.252 | *λ*_min_= 0.183 | *λ*_min_= 0.045 | *λ*_1se_= 0.211 | *λ*_min_= 0.096 | *λ*= 0.255 | *λ*= 0.305 | *λ*= 0.166 | *λ*_min_= 0.278 | *λ*= 0.423 |
| (Intercept) | -12.111 | -6.252 | -0.338 | -0.809 | 2.080 | -1.492 |  |  |  |  |  |  |
| CD34 | 1.267 | 0.485 |  |  |  |  |  |  |  |  |  |  |
| DIPK1C | 2.175 | 1.018 |  |  |  |  |  |  |  |  |  |  |
| MRC1 | 0.388 | 0.155 |  |  | 0.067 |  |  |  |  |  |  |  |
| IGF2BP3 | 0.092 |  |  |  | -0.010 |  | 0.073 |  |  |  |  |  |
| CERK | -0.076 |  |  |  |  |  |  |  | -0.033 |  |  |  |
| BTBD6 | -0.111 |  |  |  |  |  |  |  |  |  |  |  |
| HBG2 | 0.006 |  |  |  |  |  |  |  |  |  |  |  |
| LGALS3 | 0.082 |  |  |  |  |  |  |  |  |  |  |  |
| SLC25A39 | 0.048 |  |  |  |  |  |  |  |  |  |  |  |
| BRE-AS1 |  |  | 0.107 |  |  |  |  |  |  |  |  |  |
| DDIT4L |  |  | -0.095 |  |  |  |  |  |  |  |  |  |
| ANXA1 |  |  | 0.000 |  |  |  |  |  |  |  |  |  |
| WWC3 |  |  | -0.004 |  |  |  |  |  |  |  |  |  |
| PARP15 |  |  |  | -0.143 |  |  |  |  |  |  |  |  |
| PRXL2C |  |  |  | 0.124 |  |  |  |  |  |  |  |  |
| NPDC1 |  |  |  | 0.004 | 0.095 | 0.049 |  |  |  |  | 0.104 | 0.013 |
| CLEC14A |  |  |  | 0.029 | -0.236 |  |  |  |  |  |  |  |
| OPN3 |  |  |  | -0.018 | -0.284 |  |  |  |  |  |  |  |
| CD38 |  |  |  |  | -0.253 | -0.120 |  |  |  |  |  |  |
| KCNK12 |  |  |  |  | 0.156 | 0.027 |  |  |  |  |  |  |
| PRX |  |  |  |  | 0.272 | 0.208 |  |  |  |  |  |  |
| SMAD1 |  |  |  |  | 0.034 | 0.026 |  |  |  |  |  |  |
| ABCA1 |  |  |  |  | -0.113 |  |  |  |  |  |  |  |
| AHR |  |  |  |  | 0.052 |  | -0.220 |  |  |  |  |  |
| CTGF |  |  |  |  | 0.167 |  |  |  |  |  |  |  |
| DPEP1 |  |  |  |  | 0.000 |  |  |  |  |  |  |  |
| EFNA1 |  |  |  |  | 0.117 |  |  |  |  |  |  |  |
| F13A1 |  |  |  |  | 0.053 |  |  |  |  |  |  |  |
| GADD45A |  |  |  |  | 0.031 |  |  |  |  |  |  |  |
| HLA-DQB1 |  |  |  |  | 0.052 |  |  |  |  |  |  |  |
| IFI44L |  |  |  |  | -0.099 |  |  |  |  |  |  |  |
| ITGA6 |  |  |  |  | 0.030 |  |  |  |  |  |  |  |
| KLF9 |  |  |  |  | 0.135 |  |  |  |  |  |  |  |
| LOC105374869 |  |  |  |  | -0.009 |  |  |  |  |  |  |  |
| LPAR6 |  |  |  |  | 0.094 |  |  |  |  |  |  |  |
| MGST3 |  |  |  |  | -0.050 |  |  |  |  |  |  |  |
| NCF2 |  |  |  |  | -0.056 |  |  |  |  |  |  |  |
| NUCB2 |  |  |  |  | -0.093 |  |  |  |  |  |  |  |
| PHACTR3 |  |  |  |  | -1.301 |  |  |  |  |  |  |  |
| PHYH |  |  |  |  | -0.194 |  |  |  |  |  |  |  |
| PKIG |  |  |  |  | -0.031 |  |  |  |  |  |  |  |
| PON2 |  |  |  |  | 0.068 |  |  |  |  |  |  |  |
| RGL1 |  |  |  |  | 0.075 |  |  |  |  |  |  |  |
| RGS2 |  |  |  |  | -0.037 |  |  |  |  |  |  |  |
| SCN3A |  |  |  |  | 0.028 |  | 0.041 |  |  |  |  |  |
| SPRY2 |  |  |  |  | 0.110 |  |  |  |  |  |  |  |
| TIAM2 |  |  |  |  | 0.025 |  |  |  |  |  |  |  |
| TMSB15A |  |  |  |  | -0.001 |  |  |  |  |  |  |  |
| TNF |  |  |  |  | -0.194 |  |  |  |  |  |  |  |
| ZNF331 |  |  |  |  | -0.092 |  |  |  |  |  |  |  |
| PTGER2 |  |  |  |  |  | 0.008 |  |  |  |  |  |  |
| FAM241A |  |  |  |  |  |  | -0.296 | -0.016 |  |  |  |  |
| MGME1 |  |  |  |  |  |  | -0.236 | -0.024 |  |  |  |  |
| ARHGAP29 |  |  |  |  |  |  | 0.015 |  |  |  |  |  |
| BTG3 |  |  |  |  |  |  | -0.023 |  |  |  |  |  |
| CAPG |  |  |  |  |  |  | -0.190 |  |  |  |  |  |
| CYB5R2 |  |  |  |  |  |  | -0.114 |  |  |  |  |  |
| DYSF |  |  |  |  |  |  | 0.000 |  |  |  |  |  |
| FAM171A1 |  |  |  |  |  |  | -0.100 |  |  |  |  |  |
| FKBP11 |  |  |  |  |  |  | -0.116 |  |  |  |  |  |
| GOLGA8A |  |  |  |  |  |  | -0.065 |  |  |  |  |  |
| IRX1 |  |  |  |  |  |  | 0.075 |  |  |  |  |  |
| MX2 |  |  |  |  |  |  | 0.154 |  |  |  |  |  |
| SESN1 |  |  |  |  |  |  | -0.029 |  |  |  |  |  |
| SHANK3 |  |  |  |  |  |  | 0.004 |  |  |  |  |  |
| SLC15A4 |  |  |  |  |  |  | -0.043 |  |  |  |  |  |
| SNTG2-AS1 |  |  |  |  |  |  | -0.061 |  |  |  |  |  |
| SOCS5 |  |  |  |  |  |  | -0.051 |  |  |  |  |  |
| STAT1 |  |  |  |  |  |  | -0.058 |  |  |  |  |  |
| TCFL5 |  |  |  |  |  |  | 0.412 |  |  |  |  |  |
| C1QTNF4 |  |  |  |  |  |  |  |  |  | 0.026 |  |  |
| SCML1 |  |  |  |  |  |  |  |  |  | 0.006 |  |  |
| GPM6B |  |  |  |  |  |  |  |  |  |  | -0.010 |  |

**Data S1: The weights of the genes used in NMF**
